# Analysis and Identification of Candidate Genes Associated with M1 Macrophage Infiltration and Glycolysis in Breast Cancer

**DOI:** 10.1111/cbdd.70437

**Published:** 2026-11-05

**Authors:** Liang Zhang, Lingyuan Wu, Can Xu, Chao Fang, Xingsong Tian, Kunbing Zhu

**Affiliations:** ^1^ Department of Breast and Thyroid Surgery Shandong Provincial Maternal and Child Health Care Hospital Affiliated to Qingdao University Jinan Shandong China; ^2^ Reproductive Medicine Center, Shandong Provincial Maternal and Child Health Care Hospital Affiliated to Qingdao University Jinan Shandong China; ^3^ Department of Postpartum Rehabilitation Shandong Provincial Maternal and Child Health Care Hospital Affiliated to Qingdao University Jinan Shandong China; ^4^ School of Medicine Shandong University Jinan Shandong China; ^5^ Department of Breast and Thyroid Surgery Shandong Provincial Hospital Jinan Shandong China

**Keywords:** breast cancer, diagnostic biomarkers, glycolysis, M1 macrophages, Mendelian randomization, transcriptomics

## Abstract

Macrophage polarization and glycolytic reprogramming contribute to the breast cancer microenvironment. However, the genes associated with these features and their clinical relevance remain poorly defined. Breast cancer transcriptomic datasets were analyzed using immune deconvolution, single‐sample gene set enrichment analysis, and weighted gene coexpression network analysis. We identified 101 genes shared by the M1 macrophage‐associated, glycolysis‐related, and differentially expressed gene sets. Eight genes showed suggestive genetically predicted associations with BC risk. Machine learning selected seven consensus genes. In TCGA‐BRCA, six genes differed significantly between tumor and normal tissue, but only five changed in the same direction as in GSE65194. In the 56 paired clinical tissues, ADAM15, PPP1R14B, and XPO5 were upregulated in tumors, whereas FAM172A and PPM1M were downregulated. ADAM15, PPP1R14B, and XPO5 were preferentially expressed in M1‐like TAMs, whereas FAM172A and PPM1M showed higher expression in M2‐like TAMs. In BMDMs, overexpression of ADAM15, XPO5, or PPP1R14B, as well as knockdown of PPM1M or FAM172A, increased the proportion of CD80‐positive cells and ATP, glucose uptake, and lactate production. Five genes showed significant associations with overall survival. Functional enrichment analyses implicated immune regulation, extracellular matrix organization, and metabolic pathways.

## Graphical abstract

The GEO and TCGA datasets were sampled from bulk transcriptomic data and integrated with immune cell deconvolution (CIBERSORT and xCell)‐weighted gene coexpression network analysis (WGCNA) and a differential expression profile to identify the genes involved in both M1 macrophage infiltration and glycolytic activity. Interrogation of putative causal effects on the risk of breast cancer was carried out via Mendelian randomization (MR). LASSO penalized regression and support vector machine recursive feature elimination (SVM‐RFE) were used to prioritize candidate diagnostic biomarkers, which were then validated in independent cohorts and evaluated via Kaplan–Meier survival curves to determine their prognostic value. Quantitative real‐time PCR was conducted to verify the patterns of ADAM15, PPM1M, and XPO5 expression in the tumors.

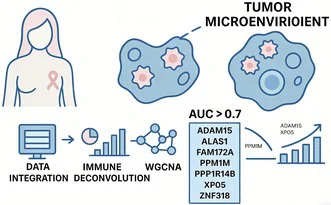

## 1 Introduction

Breast cancer (BC) continues to be the most frequently diagnosed cancer in women worldwide and remains a major cause of cancer‐related mortality. In 2022, approximately 2.3 million new breast cancer cases were estimated worldwide, making breast cancer the most commonly diagnosed cancer among women (Bray et al. 2024). Although advances in early detection, molecular characterization, and systemic treatment have improved breast cancer management, clinical outcomes remain uneven because of marked disease heterogeneity and the adaptive, often immunosuppressive tumor microenvironment (TME) (Zou et al. 2023). The TME comprises malignant cells, stromal elements, extracellular components, and infiltrating immune cells, which interact to influence tumor behavior (Kudelova et al. 2022). Among these components, macrophages are important regulators of tumor–immune interactions.

Within this context, tumor‐associated macrophages (TAMs) represent one of the most abundant immune‐cell populations in solid tumors (Mao et al. 2021; Pan et al. 2020). They exert complex and sometimes opposing effects on tumor development and progression (Gao et al. 2022; Li et al. 2021). Owing to their phenotypic plasticity, TAMs can adopt different functional states in response to local microenvironmental signals (Gao et al. 2022). M1‐like macrophages are generally associated with pro‐inflammatory cytokine production and antitumor immune activity (Wang et al. 2024), whereas M2‐like macrophages are more often linked to immunosuppression, tissue remodeling, and tumor growth (Zhang and Sioud 2023). However, TAMs do not form two fixed and mutually exclusive populations. Instead, they may display intermediate or mixed phenotypes depending on their spatial and metabolic context. The M1/M2 framework therefore describes broad functional tendencies rather than a strict binary classification (Boutilier and Elsawa 2021; Chen et al. 2022; Wu et al. 2020). Nevertheless, a higher ratio of M1‐like to M2‐like macrophage features has been associated with more favorable outcomes in several malignancies, including BC (Kuroda et al. 2021). Together, these observations support the clinical relevance of macrophage state in BC while underscoring the need to distinguish macrophage‐associated signals from transcripts contributed by other cell types in bulk tumor tissue.

Building upon this functional diversity, recent studies have highlighted the metabolic basis of macrophage activation. Classically activated M1‐like macrophages commonly exhibit increased aerobic glycolysis, with glycolytic activity remaining elevated despite the presence of oxygen (Li et al. 2023). This metabolic program may support rapid ATP production and the generation of metabolic intermediates required for inflammatory responses and is influenced by hypoxia‐inducible factor 1 alpha (HIF‐1α) and the PI3K/Akt/mTOR pathway (Zhuang et al. 2021; Kim et al. 2022; Zeng et al. 2024). M2‐like macrophages are often associated with greater use of oxidative phosphorylation and fatty acid oxidation, although these metabolic pathways are not exclusive to a single macrophage state (Fan et al. 2022). BC cells also frequently exhibit enhanced glycolytic activity, a characteristic of the Warburg phenotype that may increase lactate accumulation and contribute to acidification of the TME (Damaghi et al. 2021). Lactate‐rich conditions can suppress cytotoxic immune functions and alter macrophage behavior (Naik and Decock 2020). These observations suggest that tumor‐cell and macrophage metabolism may be functionally coupled within the TME.

Despite growing interest in tumor immunometabolism, the genes linking tumor glycolysis to M1‐like macrophage programs in BC remain poorly defined. Although tumor glycolysis and immune‐cell infiltration have each been associated with BC progression (Oshi et al. 2023; Wang et al. 2023), the gene‐level interface between these processes in M1‐like macrophages remains unclear. In addition, bulk tissue analyses cannot readily determine whether altered expression arises from tumor cells, macrophage subsets, or both. Thus, it remains unclear which genes are jointly associated with glycolytic activity and M1‐like macrophage infiltration, whether they are enriched in tumor cells or macrophage subsets, and whether these associations correspond to macrophage polarization, glycolysis‐related activity, or clinical outcome.

Accordingly, we hypothesized that genes jointly associated with glycolysis and M1‐like macrophage infiltration may mark an immune‐metabolic state associated with BC status and clinical outcome. To address this hypothesis, we integrated transcriptomic profiling, immune‐cell deconvolution, co‐expression analysis, MR‐based genetic prioritization, and machine‐learning feature selection. Selected candidates were evaluated in public cohorts, paired clinical tissues, separated primary tumor cells, M1‐like and M2‐like TAMs, and BMDMs. We aimed to identify glycolysis‐associated genes linked to M1‐like macrophage programs in BC, assess their diagnostic and survival‐related associations, and examine their cellular distribution and effects on macrophage polarization and glycolysis‐related readouts. MR findings were used for candidate prioritization and were interpreted as hypothesis‐generating rather than as proof of biological causality.

## 2 Materials and Methods

### 2.1 Data Acquisition and Preprocessing

Gene‐expression profiles and sample annotations for GSE65194 were obtained from the Gene Expression Omnibus (GEO) database. The dataset included 130 bc samples and 11 adjacent normal breast samples. RNA‐sequencing data and available clinical information for the TCGA‐BRCA cohort were obtained from The Cancer Genome Atlas (TCGA), including 1095 bc samples and 113 normal breast samples. The GSE65194 microarray data and TCGA‐BRCA RNA‐sequencing data were processed and normalized separately. Expression values were log_2_‐transformed when necessary and normalized within each cohort. The two datasets were not combined into a single expression matrix. GSE65194 was used for discovery analyses, whereas TCGA‐BRCA was used for external expression validation and exploratory survival analyses. Available baseline clinicopathological characteristics for GSE65194, TCGA‐BRCA, and the 56‐patient hospital cohort are summarized in Table S1.

### 2.2 Immune Cell Infiltration Estimation

The relative proportions of 22 immune‐cell types were estimated using the CIBERSORT algorithm. The xCell algorithm was used to calculate M1 macrophage enrichment scores. Differences in immune‐cell levels between tumor and normal groups were evaluated using the Wilcoxon rank‐sum test. Correlations among immune‐cell populations were assessed using Spearman's rank correlation analysis.

### 2.3 Weighted Gene Co‐Expression Network Analysis (WGCNA)

WGCNA was performed using xCell‐derived M1 macrophage scores and ssGSEA‐derived glycolysis scores as phenotypic traits. The soft‐thresholding power was selected according to the scale‐free topology fit and mean connectivity. Genes were grouped into co‐expression modules by hierarchical clustering and dynamic tree cutting. Module membership (MM) was defined as the correlation between gene expression and the corresponding module eigengene, and gene significance (GS) represented the correlation between gene expression and the relevant phenotypic trait. Modules most strongly associated with M1 macrophage abundance or glycolysis scores were selected for subsequent analysis. Genes with MM > 0.3 and GS > 0.3 were considered M1 macrophage‐associated, whereas MM > 0.8 and GS > 0.5 were used to identify glycolysis‐associated genes.

### 2.4 Differential Expression and Enrichment Analysis

Differentially expressed genes (DEGs) between BC and normal tissues were identified using the limma package. Genes with an absolute log_2_ fold change > 1 and an adjusted *p* value < 0.05 were considered differentially expressed. Functional enrichment analysis was performed using clusterProfiler for Gene Ontology (GO) and Kyoto Encyclopedia of Genes and Genomes (KEGG) pathways. *p* values were adjusted using the Benjamini–Hochberg method.

### 2.5 Single‐Sample Gene Set Enrichment Analysis (ssGSEA)

The REACTOME_GLYCOLYSIS gene set from the Molecular Signatures Database was used to calculate sample‐level glycolysis scores by ssGSEA. Genes from glycolysis‐associated modules were intersected with M1 macrophage‐associated genes and DEGs to define glycolysis‐related M1 macrophage‐associated DEGs (MMG‐DEGs).

### 2.6 MR Analysis

Two‐sample MR analysis was performed using the TwoSampleMR package to examine the associations between genetically predicted expression of the MMG‐DEGs and BC risk. Expression quantitative trait loci from the Genotype‐Tissue Expression (GTEx) project were used as genetic instruments, and genetic association data for BC were obtained from the genome‐wide association study (GWAS) dataset ebi‐a‐GCST90018799. Single‐nucleotide polymorphisms (SNPs) with *p* < 5 × 10^−6^ were selected as instrumental variables and clumped using an *R* ^2^ threshold < 0.001. Instrumental variables with an F‐statistic > 10 were retained. The inverse‐variance weighted (IVW) method was used as the primary analysis, with MR‐Egger regression and the weighted‐median method used for sensitivity analyses.

### 2.7 Machine‐Learning Feature Selection

LASSO logistic regression was performed using the glmnet package, with tumor and normal tissue status as the classification outcome. Ten‐fold cross‐validation was used to select the penalty parameter corresponding to the minimum binomial deviance. SVM‐RFE was performed using the e1071 package. Genes selected by both methods were retained as consensus diagnostic candidates.

### 2.8 Diagnostic Model Construction and Evaluation

The diagnostic performance of the selected genes was evaluated using receiver operating characteristic (ROC) curve analysis implemented in the pROC package. The area under the curve was used to assess the ability of each gene to distinguish BC from non‐cancer samples. ROC analysis was also performed using the qRT‐PCR data from the 56‐patient cohort to assess how well each of the five genes distinguished tumor tissues from paired adjacent non‐tumor tissues. A gene‐based nomogram was constructed using the rms package. Decision curve analysis was used to assess the net benefit of the model across a range of threshold probabilities. Calibration plots compared predicted and observed probabilities and bias‐corrected calibration was assessed using bootstrap resampling.

### 2.9 Expression Validation and Survival Analysis

Expression differences were evaluated independently in the GSE65194 and TCGA‐BRCA cohorts. Associations between candidate‐gene expression and M1 macrophage scores were assessed using Pearson's correlation analysis. Overall survival was evaluated using Kaplan–Meier analysis and the log‐rank test. Patients were divided into high‐ and low‐expression groups according to the median expression level of each gene. Univariate and multivariable Cox proportional‐hazards regression analyses were performed for the candidate genes evaluated in the survival analysis. Candidate‐gene expression variables were entered simultaneously into the multivariable model. Hazard ratios and corresponding 95% confidence intervals were calculated, and the proportional‐hazards assumption was assessed using Schoenfeld residuals.

### 2.10 Regulatory Network Construction

Potential microRNA (miRNA)–messenger RNA (mRNA) interactions were obtained from miRTarBase, TargetScan, and StarBase. Candidate transcription factors (TFs) were identified using information from Enrichr, TRANSFAC, and JASPAR. The predicted miRNA–TF–mRNA relationships involving the selected hub genes were integrated and visualized in Cytoscape.

### 2.11 Gene Set Enrichment Analysis (GSEA)

GSEA was performed separately for each selected hub gene. Samples were divided into high‐ and low‐expression groups according to the median expression level of the corresponding gene. KEGG pathway gene sets were used for enrichment analysis, and adjusted *p* values were used to evaluate pathway significance.

### 2.12 Clinical Tissue Specimens

A total of 56 patients with pathologically confirmed BC who underwent surgery were enrolled in this study. Paired tumor and adjacent nontumor tissues were collected during surgery, immediately snap‐frozen in liquid nitrogen, and stored at −80°C until further analysis. The study was approved by the Research Ethics Committee of Shandong Province Maternal and Child Health Care Hospital (No. 2025‐031). Written informed consent was obtained from all 56 participants before tissue collection.

### 2.13 Isolation of Primary Tumor Cells and Tumor‐Associated Macrophage Subsets

Fresh breast tumor specimens were processed shortly after collection. The tissues were minced into small fragments and incubated in RPMI‐1640 containing collagenase IV (1 mg/mL), hyaluronidase (0.1 mg/mL), and DNase I (50 μg/mL) at 37°C for 30 min with gentle agitation. The digested tissue was passed through a 70‐μm cell strainer and treated with an ammonium chloride‐based erythrocyte lysis buffer. After washing with PBS, the cells were resuspended in PBS containing 2% fetal bovine serum and 1 mM EDTA. The cell suspension was labeled with fluorochrome‐conjugated antibodies against EpCAM, CD45, CD11b, CD80, CD86, CD163, and CD206. Tumor cells were defined as EpCAM‐positive and CD45‐negative cells. Tumor‐associated macrophages were identified within the CD45‐positive CD11b‐positive myeloid fraction. M1‐like TAMs were defined by the co‐expression of CD80 and CD86, whereas M2‐like TAMs were defined by the co‐expression of CD163 and CD206. CD68 staining was performed on a parallel aliquot to confirm macrophage identity. The indicated cell populations were separated by fluorescence‐activated cell sorting, and the purity of each fraction was assessed by flow cytometry. Cell fractions with a purity exceeding 90% were used for RNA extraction. The isolated tumor cells, M1‐like TAMs, and M2‐like TAMs were analyzed for ADAM15, PPP1R14B, XPO5, FAM172A, and PPM1M expression by qRT‐PCR.

### 2.14 Bone Marrow‐Derived Macrophage Culture and Gene Manipulation

Bone marrow cells were collected from the femurs and tibias of 6–8‐week‐old C57BL/6 mice and passed through a 70‐μm cell strainer. After erythrocyte removal, the cells were cultured in complete DMEM supplemented with 10% fetal bovine serum, 1% penicillin–streptomycin, and recombinant macrophage colony‐stimulating factor (M‐CSF; 20 ng/mL). The culture medium was partially replaced on day 3, and macrophages were obtained after 7 days of differentiation. The differentiated BMDMs were replated for gene‐manipulation experiments. ADAM15, XPO5, and PPP1R14B were overexpressed using lentiviral vectors, whereas PPM1M and FAM172A were silenced using lentiviral short‐hairpin RNA vectors. Empty‐vector and nontargeting short‐hairpin RNA groups were processed in parallel. The cells were analyzed 48 h after gene delivery.

### 2.15 Assessment of Macrophage Polarization and Glycolysis‐Related Parameters

CD80 expression was measured by flow cytometry using a fluorescein isothiocyanate‐conjugated anti‐CD80 antibody. The percentage of CD80‐positive cells was calculated from the corresponding flow‐cytometry gates. Intracellular ATP levels were determined using a commercial luciferase‐based ATP assay kit. Glucose uptake was measured using the fluorescent glucose analogue 2‐NBDG. Lactate production was quantified using a commercial enzymatic lactate assay kit. The results were normalized to total protein content.

### 2.16 RNA Isolation and qRT‐PCR Analysis

Total RNA was extracted from tumor tissues, adjacent nontumor tissues, separated primary tumor cells, M1‐like TAMs, and M2‐like TAMs using the Total RNA Extraction Kit (Cat. No. R1200, Solarbio, Beijing, China). Complementary DNA was synthesized using the Universal RT‐PCR Kit (M‐MLV; Cat. No. RP1100, Solarbio). Quantitative real‐time PCR was performed with 2× SYBR Green PCR Master Mix (Cat. No. SR1110, Solarbio) on an ABI 7500 Real‐Time PCR Detection System (Applied Biosystems, USA). The expression levels of ADAM15, PPP1R14B, XPO5, FAM172A, and PPM1M were normalized to GAPDH. Relative expression was calculated using the 2^−ΔΔCt^ method.

### 2.17 Clinicopathological Association Analysis

For the 56‐patient tissue cohort, patients were divided into high‐ and low‐expression groups according to the median expression level of each candidate gene. Associations between gene‐expression groups and available clinicopathological variables, including age, clinical stage, estrogen receptor (ER) status, progesterone receptor (PR) status, and human epidermal growth factor receptor 2 (HER2) status, were assessed using the chi‐square test or Fisher's exact test, as appropriate.

### 2.18 Statistical Analysis

All statistical analyses were performed in R version 4.2.2. Student's *t*‐test was used for comparisons between two groups, with a paired *t*‐test applied to matched tumor and adjacent nontumor tissues. One‐way analysis of variance with post hoc multiple comparisons was used for comparisons among three or more groups. Categorical variables were compared using the chi‐square test or Fisher's exact test. Kaplan–Meier curves were compared using the log‐rank test, and Cox regression was used for survival analysis. *p* < 0.05 was considered statistically significant. Cell‐based experiments were performed using three independent biological replicates.

## 3 Results

### 3.1 Composition of Tumor‐Infiltrating Immune Cells and Identification of M1 Macrophage–Associated Genes

CIBERSORT analysis of 22 immune cell types in BC and adjacent normal tissues revealed marked differences in immune‐cell composition between the two groups, indicating remodeling of the tumor immune microenvironment (Figure 1A). The strongest positive correlation was observed between CD8^+^ T cells and activated CD4^+^ memory T cells (*r* = 0.46), whereas M1 and M2 macrophages showed the strongest negative correlation (*r* = −0.48) (Figure 1B). In total, 10 immune‐cell populations differed significantly between BC and normal tissues, with M1 macrophages displaying the largest increase in tumors (Figure 1C). Using xCell‐derived M1 macrophage abundance as the phenotypic trait, WGCNA identified the turquoise module as the module most closely related to M1 macrophage infiltration (Figure 1D). Applying the criteria of MM > 0.3 and GS > 0.3 yielded 2374 M1 macrophage–associated genes (Figure 1E).

**FIGURE 1:**
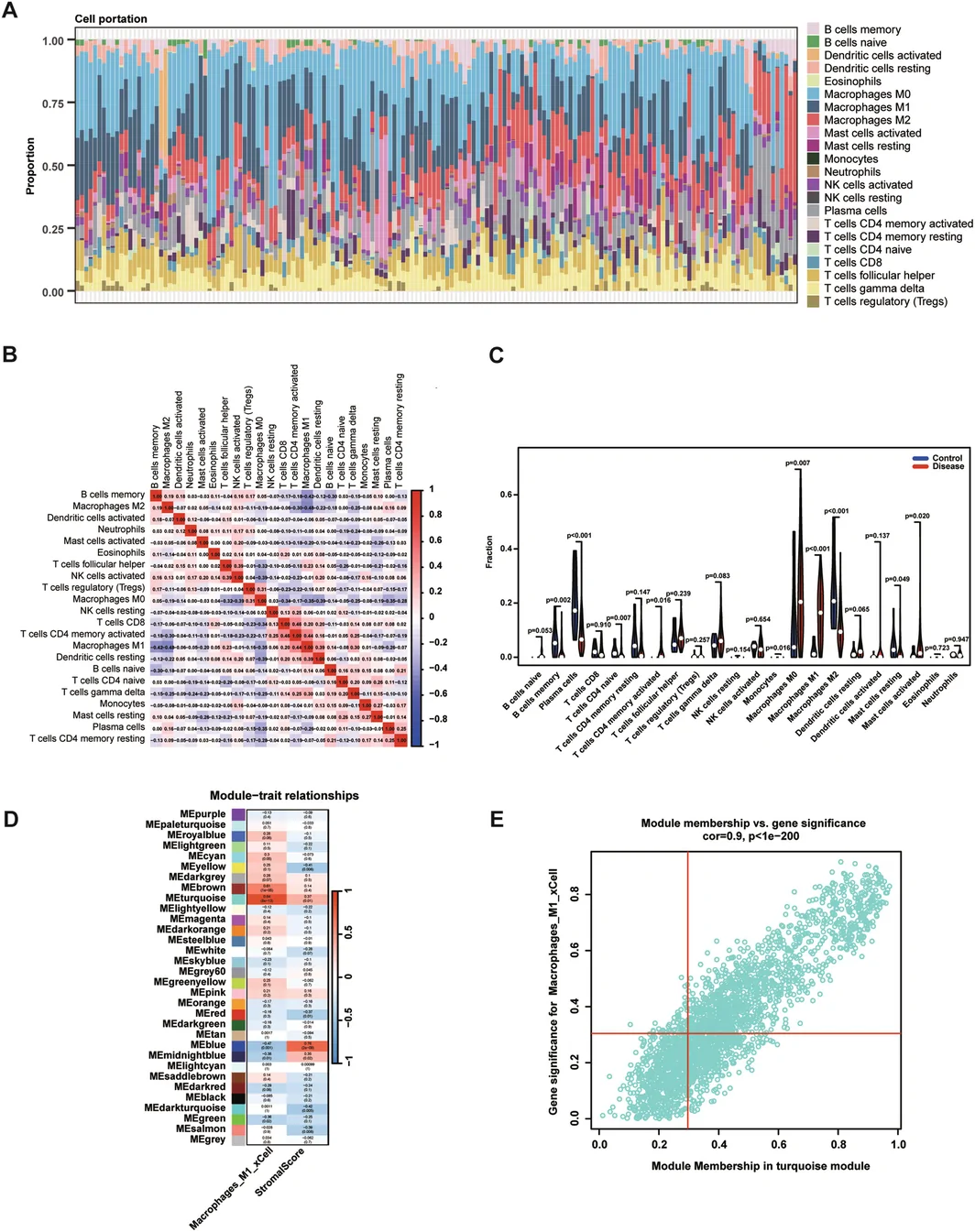
Composition of tumor‐infiltrating immune cells and identification of M1 macrophage‐associated genes in BC. (A) A stacked bar chart displays the estimated proportions of 22 distinct immune cell types in BC tissues and adjacent normal breast samples, as inferred using the CIBERSORT method. (B) A heatmap illustrates the pairwise Spearman correlation coefficients among the infiltrating immune cell populations across all samples. (C) Violin plots visualize the distributions of immune infiltration levels between the tumor and normal groups across individual immune cell types. (D) A heatmap derived from WGCNA illustrates the correlation strength between gene expression modules and M1 macrophage abundance. (E) A scatter plot presents the relationship between module membership values and gene significance scores within the M1 macrophage‐correlated module, which was used to identify representative genes.

### 3.2 Differential Gene Expression and Enrichment Analysis

Analysis of the GSE65194 dataset identified 3267 DEGs between BC and normal tissues, including 1662 upregulated and 1605 downregulated genes (Figure 2A,B). KEGG enrichment analysis highlighted the cell cycle, p53 signaling, glutathione metabolism, necroptosis, IL‐17 signaling, TNF signaling, and glycolysis pathways (Figure 2C). GO analysis identified chromosome segregation, mitotic nuclear division, and extracellular matrix organization as the major enriched biological processes and cellular components (Figure 2D). These results indicated concurrent alterations in proliferative activity and extracellular‐matrix organization in BC tissues.

**FIGURE 2:**
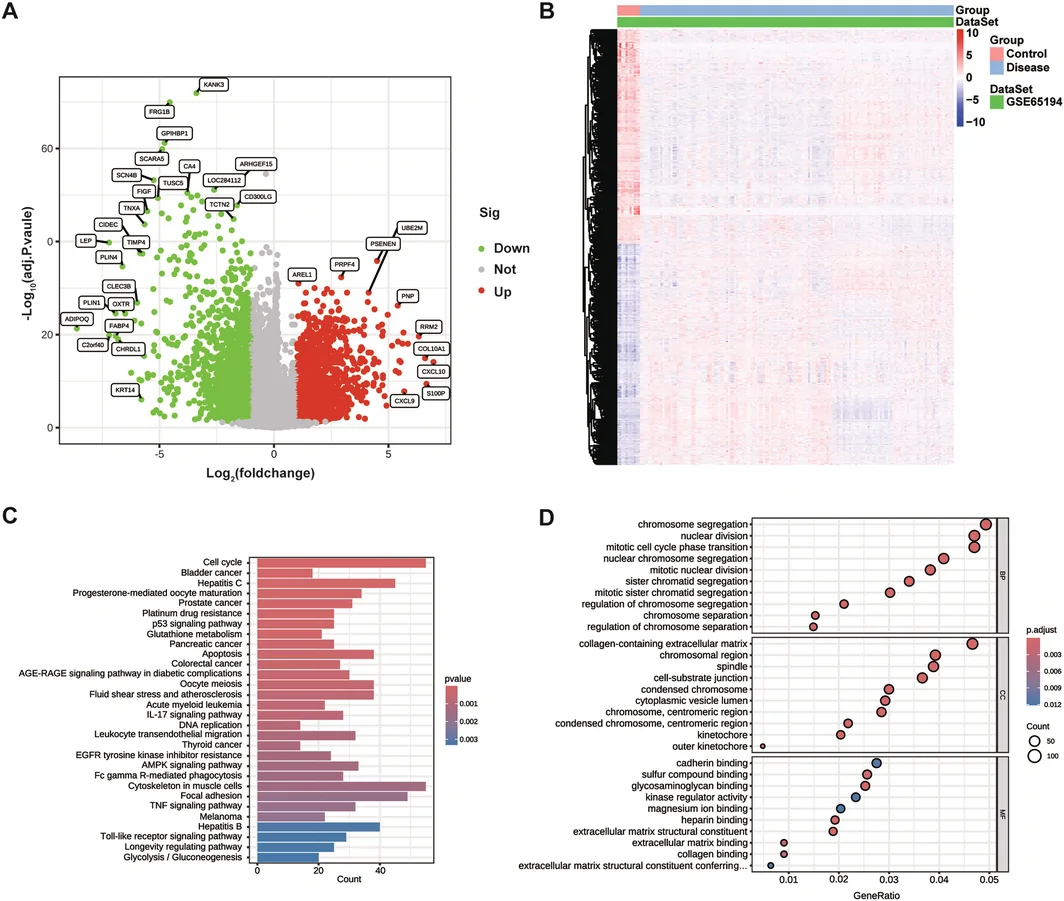
Identification of DEGs and functional enrichment analysis in BC. (A) The volcano plot depicts DEGs between tumor and normal samples in the GSE65194 dataset, with red and green dots representing upregulated and downregulated genes, respectively. (B) The heatmap illustrates the expression patterns of DEGs across all analyzed samples, highlighting transcriptional variation between the two groups. (C) The bar chart summarizes the KEGG pathways enriched among the DEGs, ranked by gene count and statistical significance. (D) The bubble plot presents the GO enrichment results for the biological process (BP), cellular component (CC), and molecular function (MF) categories, with circle size and color indicating gene counts and adjusted *p*‐values, respectively.

### 3.3 Identification of Glycolysis‐Related DEGs Associated With M1 Macrophages

The glycolysis ssGSEA score was significantly higher in BC samples than in control samples (Figure 3A). Sample clustering and outlier assessment were performed before network construction to evaluate data quality (Figure 3B). The soft‐threshold analysis identified β = 4 as the first power reaching a scale‐free topology fit of approximately 0.88 (Figure 3C). Using this parameter, WGCNA grouped genes into co‐expression modules through hierarchical clustering and dynamic tree cutting (Figure 3D). The turquoise module had the strongest relationship with the glycolysis score (Figure 3E). Applying MM > 0.8 and GS > 0.5 identified 5182 glycolysis‐associated genes (Figure 3F). Intersecting these genes with the M1 macrophage–associated genes and DEGs produced 101 overlapping MMG‐DEGs (Figure 3G).

**FIGURE 3:**
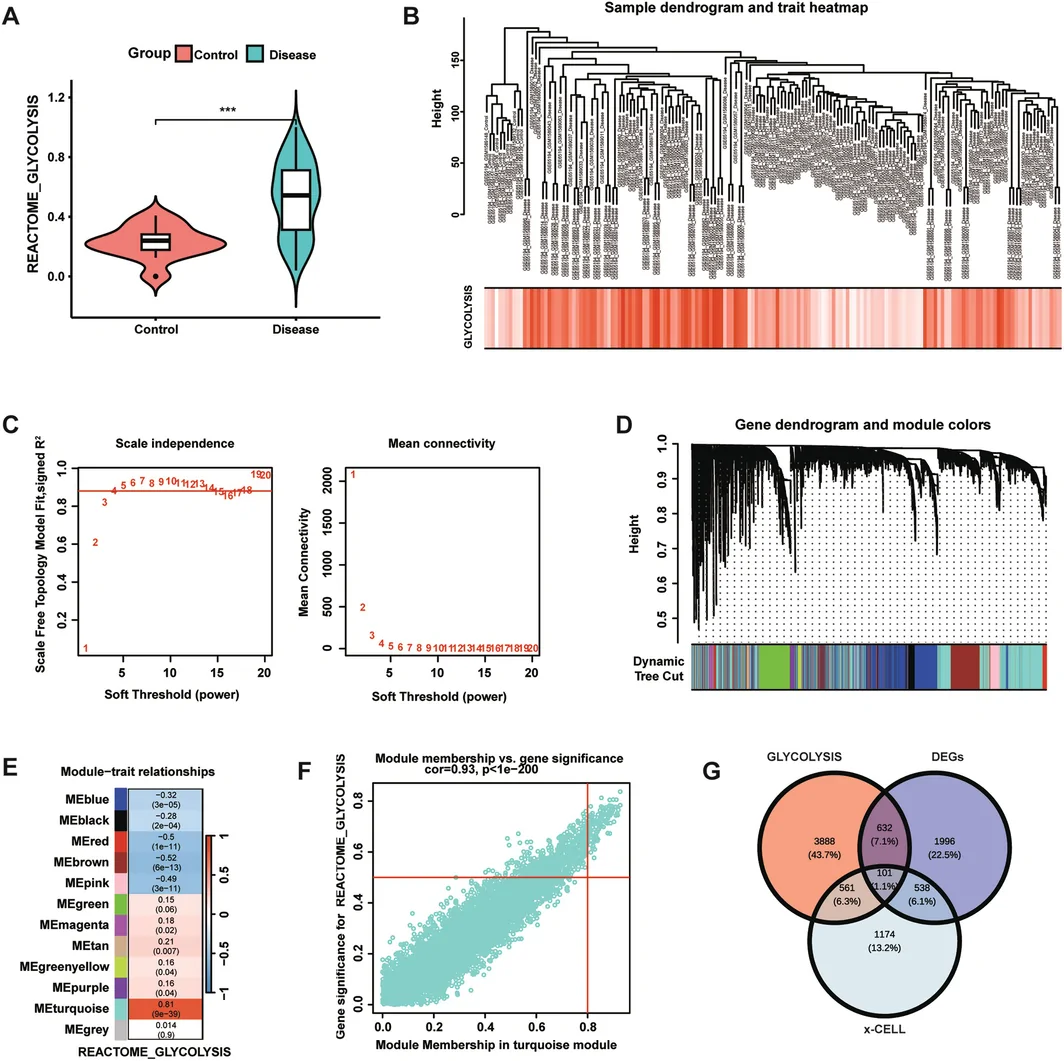
Screening of glycolysis‐related DEGs associated with M1 macrophages in BC. (A) The violin plot presents the distribution of glycolysis pathway scores in BC and control samples, as determined by ssGSEA. (B) The sample dendrogram and accompanying trait heatmap outline the hierarchical relationships and phenotype groupings before network construction. (C) The two plots assess the effects of different soft‐threshold powers on scale‐free topology and average connectivity. (D) The gene dendrogram visualizes the clustering of genes according to expression similarity, with module colors assigned by dynamic tree cutting. (E) The heatmap displays the correlations between module eigengenes (MEs) and glycolysis pathway scores across all identified modules. (F) The scatter plot evaluates the relationship between module membership and gene significance within the selected module. (G) The Venn diagram compares the gene sets derived from DEGs, xCell‐derived M1 macrophage‐associated genes, and glycolysis‐related genes to identify common elements.

### 3.4 MR Analysis of Candidate Genes

MR analysis of the 101 MMG‐DEGs identified eight genes with suggestive associations with BC: ADAM15, ALAS1, FAM172A, PPM1M, PPP1R14B, TP53BP2, XPO5, and ZNF318 (Figure 4). The MR estimate for PPM1M was positive, whereas the estimates for the other seven genes were negative.

**FIGURE 4:**
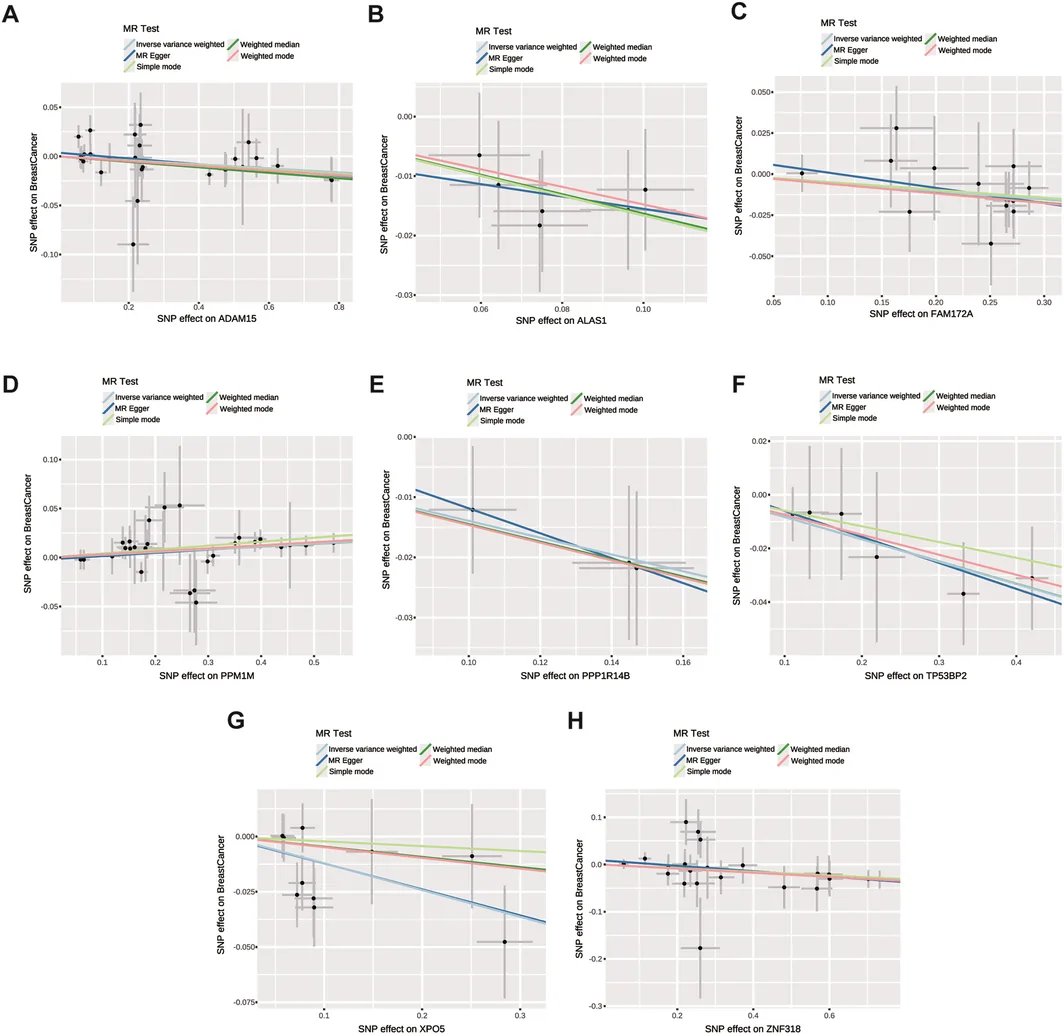
MR analysis of candidate genes in relation to BC risk. (A–H) Scatter plots depict the associations between genetic variants (SNPs) and BC risk for eight candidate genes: ADAM15, ALAS1, FAM172A, PPM1M, PPP1R14B, TP53BP2, XPO5, and ZNF318. MR estimates were obtained using the IVW, MR‐Egger, weighted median, simple mode, and weighted mode methods.

### 3.5 Selection of Diagnostic Biomarkers

LASSO regression retained seven genes at the optimal penalty value (Figure 5A,B), whereas SVM‐RFE selected eight genes (Figure 5C,D). Their intersection yielded seven consensus candidates: ADAM15, ALAS1, FAM172A, PPM1M, PPP1R14B, XPO5, and ZNF318 (Figure 5E).

**FIGURE 5:**
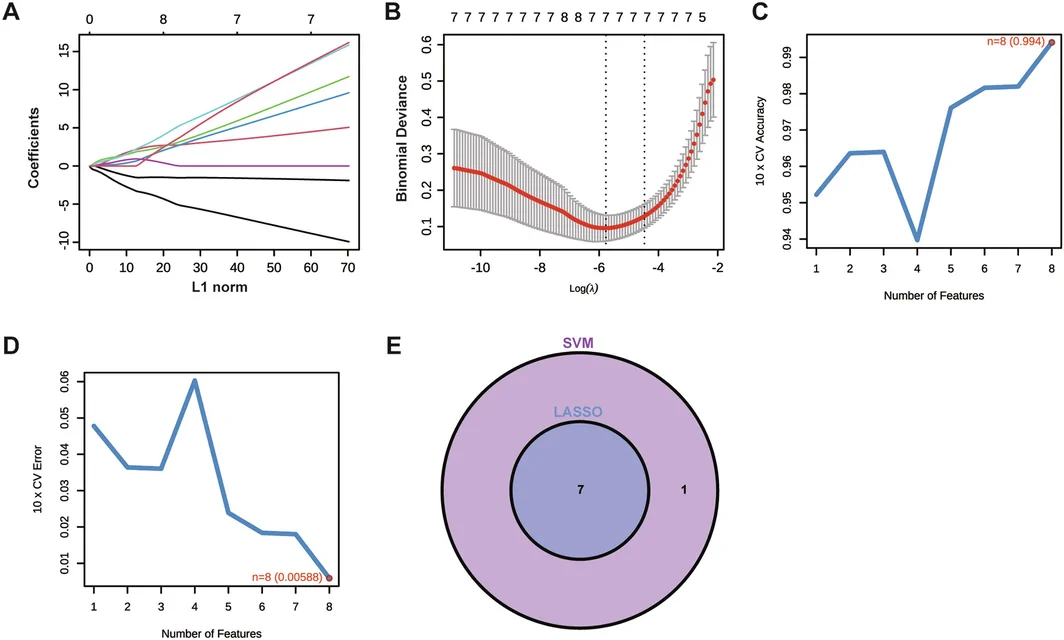
Identification of diagnostic feature genes using LASSO and SVM‐RFE algorithms. (A) The LASSO coefficient trajectories illustrate the progressive reduction in gene weights as the penalty term increases, with seven features retained at the optimal regularization level. (B) Ten‐fold cross‐validation identifies the most appropriate value of the tuning parameter (λ) in the LASSO model based on the minimum binomial deviance. (C) The line chart depicts the variation in classification accuracy across different subsets of genes selected by the SVM‐RFE algorithm, highlighting performance changes as the number of features increases. (D) The SVM‐RFE cross‐validation error plot identifies the number of features associated with the minimum prediction error and provides the basis for final gene selection. (E) The Venn diagram summarizes the overlap between the gene sets derived from LASSO and SVM‐RFE, identifying seven consensus genes shared by both machine‐learning approaches.

### 3.6 Diagnostic Performance and Model Construction

In the training dataset, all seven genes achieved an AUC greater than 0.7 for discriminating BC from non‐cancer samples (Figure 6A). A nomogram incorporating the seven genes was constructed to estimate the predicted probability of BC for individual samples based on gene‐expression levels (Figure 6B). The bias‐corrected calibration curve departed from the ideal line, particularly at lower predicted probabilities (Figure 6D). Decision curve analysis indicated a higher net benefit for the seven‐gene model than for the treat‐all or treat‐none strategies across a range of threshold probabilities (Figure 6C).

**FIGURE 6:**
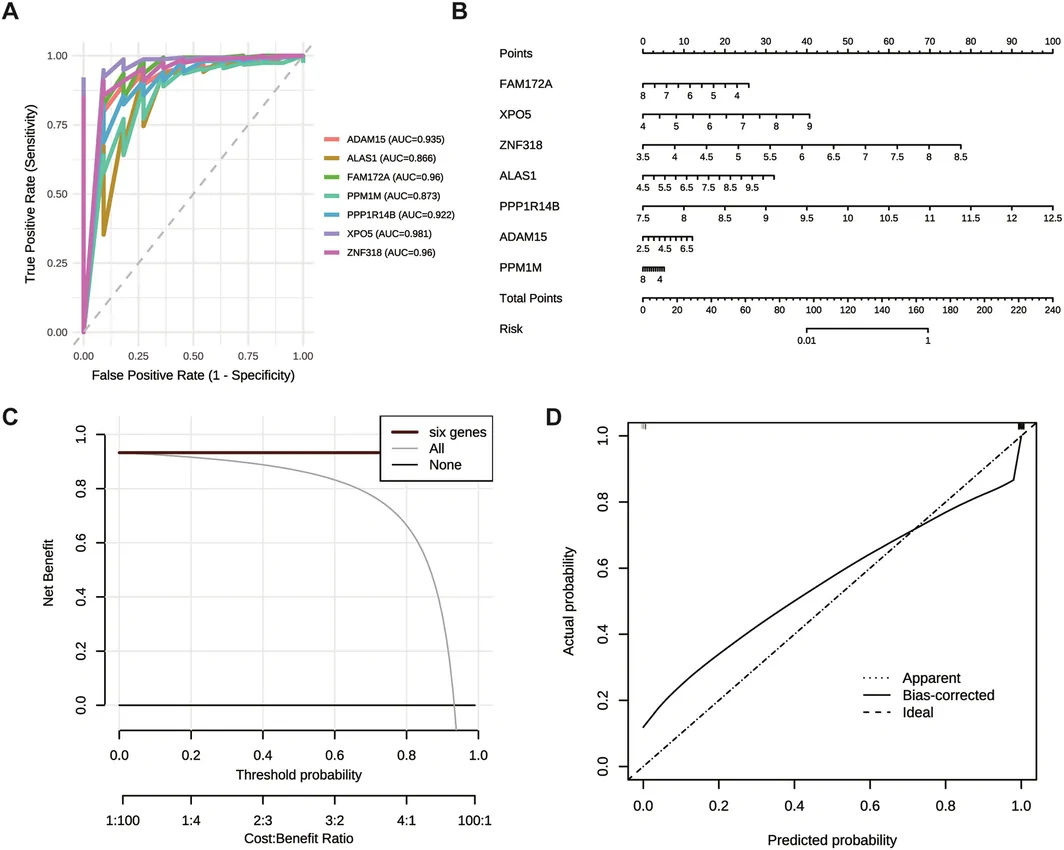
Evaluation of diagnostic performance and predictive modeling based on key genes. (A) ROC curves assess the classification performance of seven selected genes in distinguishing BC from non‐cancer samples, with all AUC values exceeding 0.7 in the training set. (B) A nomogram was constructed using the selected genes to estimate the individual probability of BC based on gene expression levels. (C) Decision curve analysis compares the net clinical benefit of the seven‐gene nomogram across various threshold probabilities with treat‐all and treat‐none strategies. (D) The calibration curve assesses the agreement between predicted probabilities and observed outcomes, with bias‐corrected and ideal curves included for reference.

### 3.7 Validation and Immune Correlation

All seven candidate genes differed significantly between BC and normal tissues in the GSE65194 dataset (Figure 7A). In TCGA‐BRCA, ADAM15, FAM172A, PPM1M, PPP1R14B, and XPO5 showed significant tumor–normal differences in the same direction as in GSE65194. ALAS1 was also significantly different, but changed in the opposite direction; ZNF318 was not significantly different (Figure 7B). Among the five genes subsequently carried forward for further analysis, ADAM15, FAM172A, PPM1M, PPP1R14B, and XPO5 were positively correlated with M1 macrophage infiltration (Figure 7C).

**FIGURE 7:**
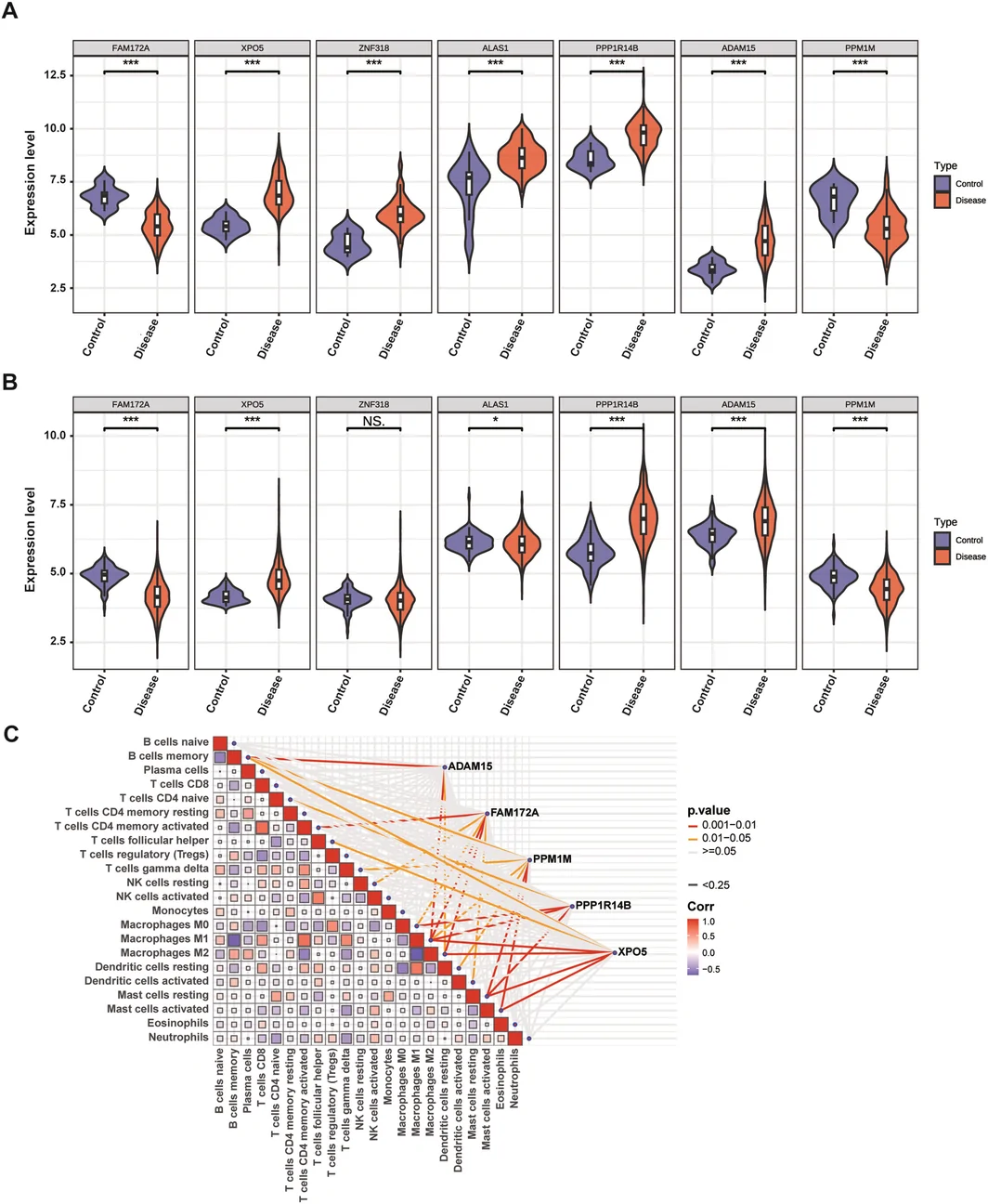
Expression validation and immune correlation analysis of selected genes in BC. (A) Violin plots compare the expression levels of seven candidate genes between cancer and normal tissues in the GSE65194 dataset. (B) The expression patterns of the same gene panel are independently assessed in the TCGA‐BRCA dataset; six genes show significant tumor–normal differences, whereas ZNF318 does not reach statistical significance. (C) Pairwise correlation analysis examines the associations between gene expression and immune‐cell proportions; five genes show positive associations with CIBERSORT‐estimated M1 macrophage infiltration. *p < 0.05, **p < 0.01, ***p < 0.001.

### 3.8 Prognostic Associations

Kaplan–Meier analysis indicated that patients with high ADAM15, FAM172A, or XPO5 expression had shorter overall survival than those with low expression. In contrast, higher PPM1M or PPP1R14B expression was associated with longer overall survival (Figure 8A–E). The high‐ and low‐expression groups were defined according to the median expression level of each gene. Based on these survival associations, ADAM15, FAM172A, PPM1M, PPP1R14B, and XPO5 were selected for further experimental validation.

**FIGURE 8:**
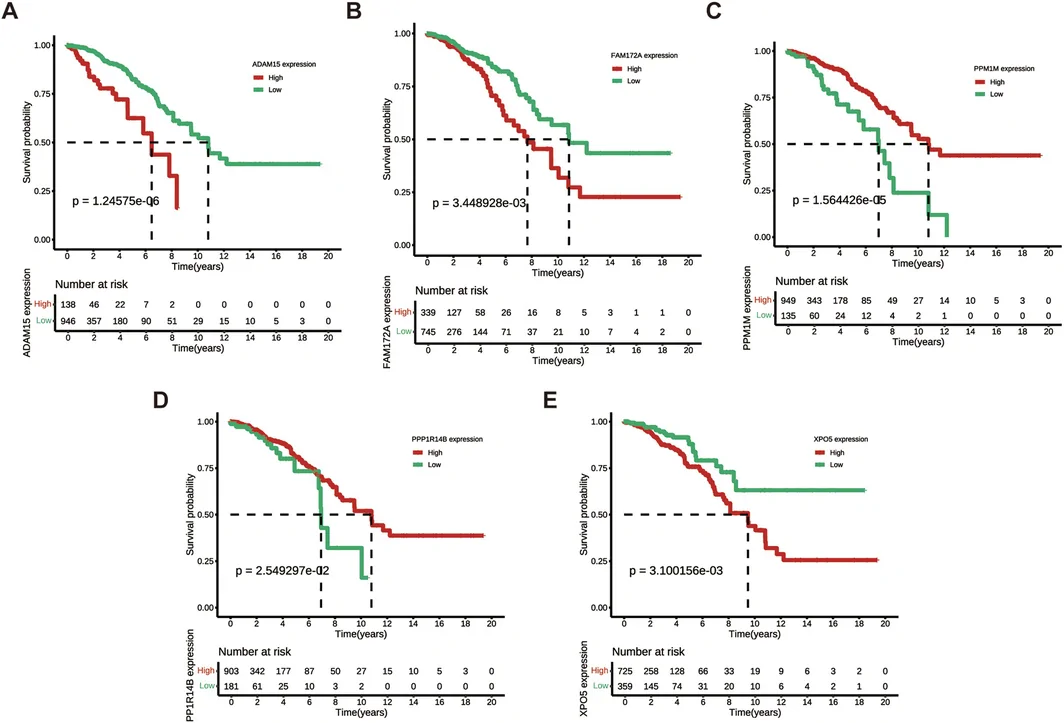
Prognostic relevance of gene expression levels in BC based on overall survival analysis. (A–E) Kaplan–Meier curves compare overall survival between patients with high and low expression of five key genes (ADAM15, FAM172A, PPM1M, PPP1R14B, and XPO5) in BC. Patients were stratified according to the median expression level, and *p*‐values were calculated using the log‐rank test.

### 3.9 Regulatory Network and Pathway Analysis

A miRNA–TF–mRNA regulatory network was constructed for the five hub genes and included 27 transcription factors and 68 miRNAs (Figure 9). Single‐gene GSEA identified distinct pathway patterns for the five genes. ADAM15 was associated with ECM–receptor interaction and focal adhesion, FAM172A with oxidative phosphorylation, PPM1M with ECM–receptor interaction, PPP1R14B with focal adhesion, and XPO5 with the TCA cycle and proteasome‐related pathways (Figure 10). The enriched pathways involved oncogenic signaling, extracellular‐matrix remodeling, and metabolic regulation.

**FIGURE 9:**
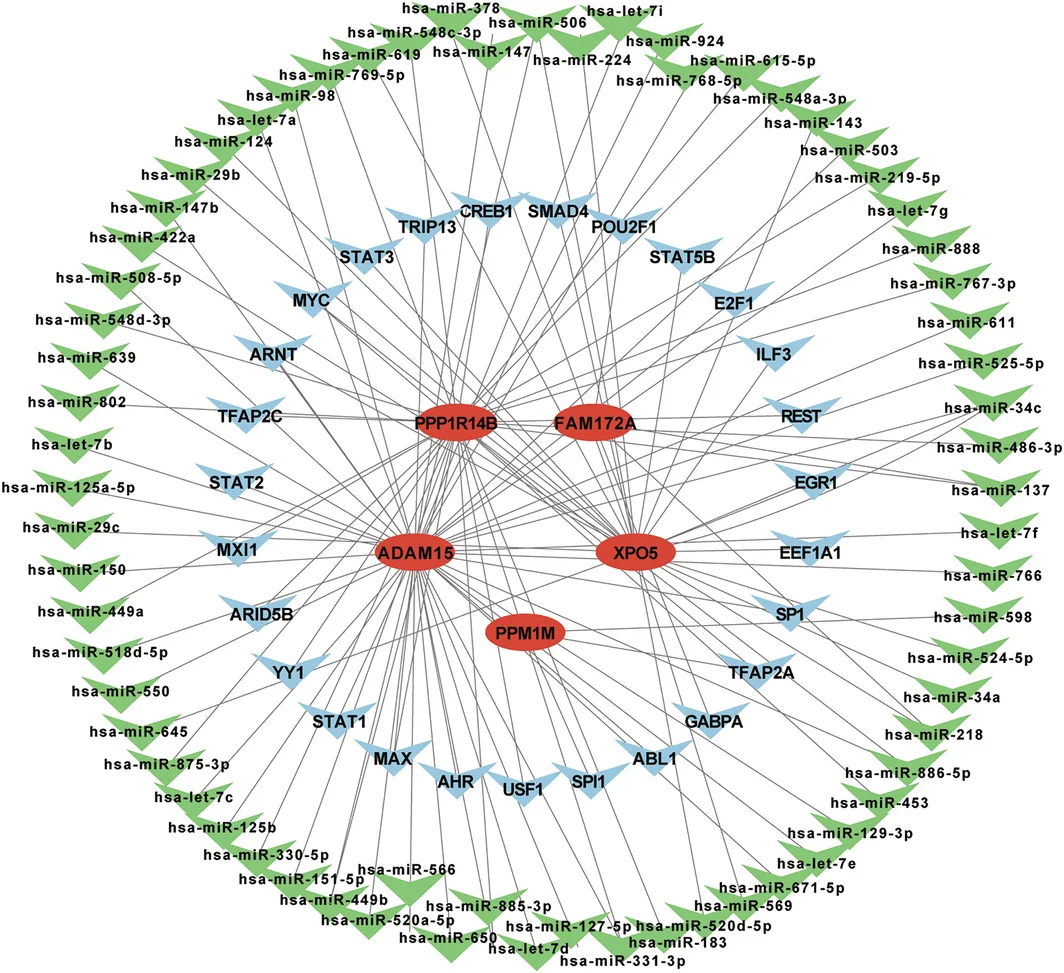
Integrated regulatory network of miRNAs, transcription factors, and key mRNAs in BC. The network diagram depicts the predicted interactions among five hub genes (red ellipses), 27 transcription factors (TFs; blue triangles), and 68 miRNAs (green diamonds), illustrating potential multilayer regulatory relationships in BC.

**FIGURE 10:**
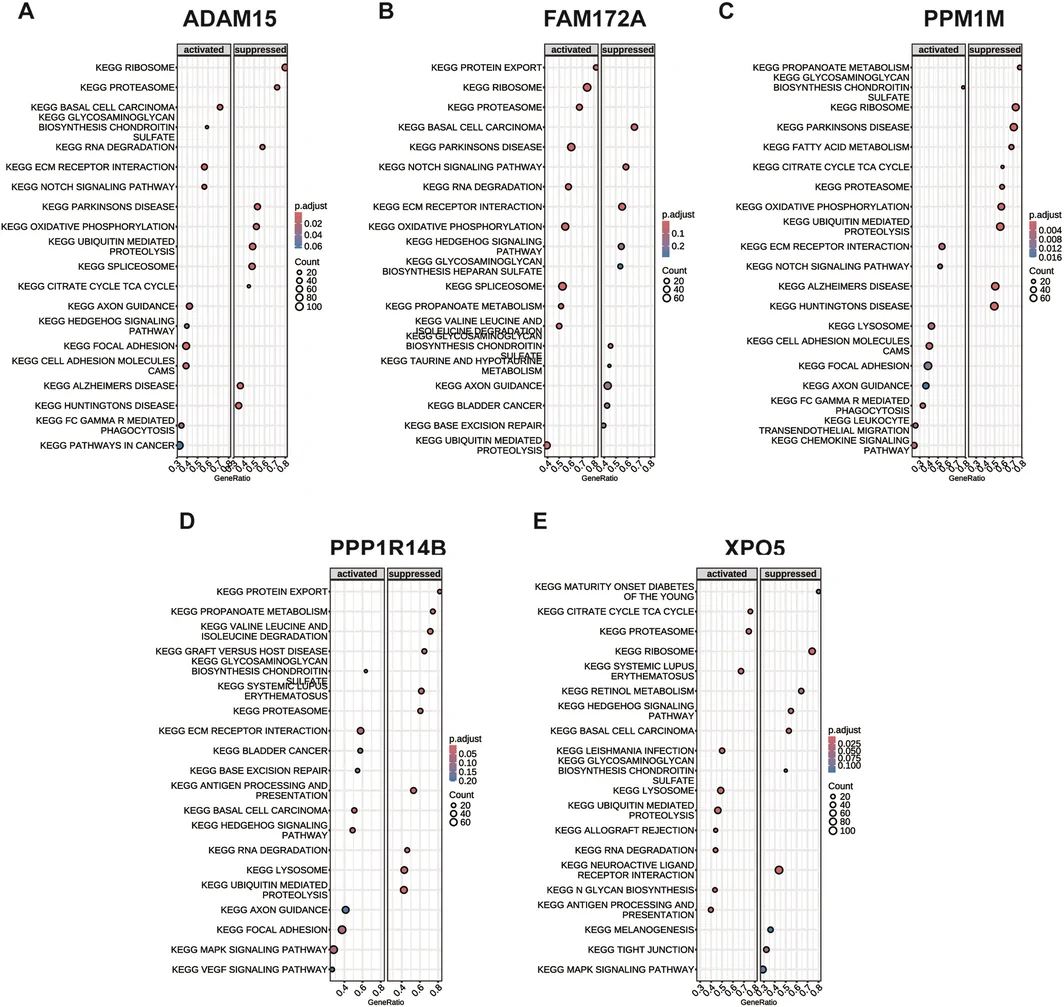
Gene set enrichment analysis of five hub genes in BC. (A–E) GSEA was performed separately for ADAM15, FAM172A, PPM1M, PPP1R14B, and XPO5 according to gene‐expression stratification. Bubble plots display the enriched KEGG pathways, categorized as activated or suppressed, with pathway significance represented by adjusted *p*‐values and gene counts.

### 3.10 Validation of Candidate‐Gene Expression in BC Tissues

To validate the bioinformatic findings in clinical specimens, qRT‐PCR was performed using paired tumor and adjacent non‐tumor tissues from 56 patients with BC. ADAM15, PPP1R14B, and XPO5 transcript levels were higher in tumor tissues, whereas FAM172A and PPM1M levels were lower than those in the paired adjacent tissues (Figure 11). ROC analysis in the same cohort yielded AUC values of 0.8559 for ADAM15, 0.6827 for PPP1R14B, 0.6014 for XPO5, 0.7286 for FAM172A, and 0.7698 for PPM1M.

**FIGURE 11:**
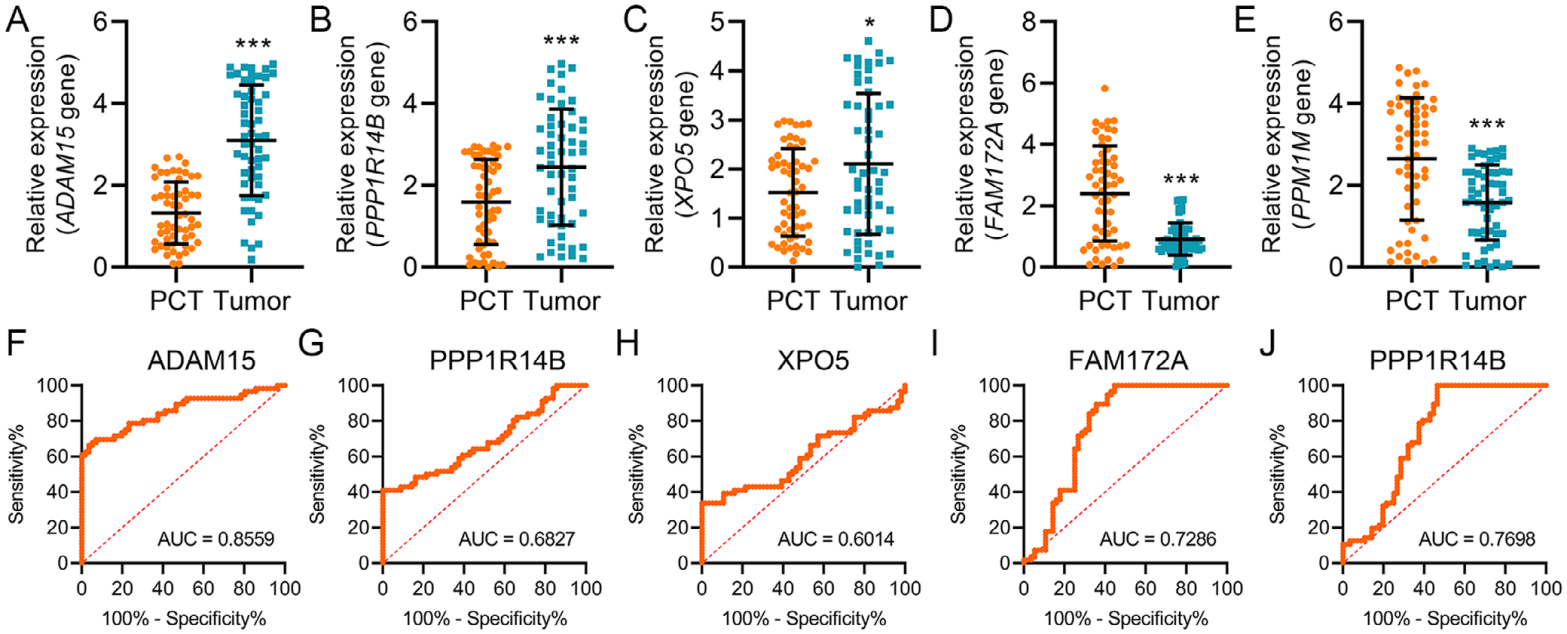
Expression of five candidate genes and their diagnostic performance in BC tissues. (A–E) Paired expression analyses compare ADAM15, PPP1R14B, XPO5, FAM172A, and PPM1M levels between tumor and adjacent non‐tumor tissues from 56 patients. (F–J) ROC curves evaluate the diagnostic performance of the five genes, with the corresponding AUC values shown in the plots.

### 3.11 Clinical Associations in the 56‐Patient Cohort

The associations between candidate‐gene expression and clinicopathological characteristics in the 56‐patient cohort are summarized in Tables 1, 2, 3, 4, 5. Thirty‐eight patients were younger than 60 years and 18 were aged 60 years or older. Forty‐six patients were ER‐positive, 42 were PR‐positive, and 17 were HER2‐positive. The cohort included 23 patients with Stage I disease, 19 with Stage II disease, and 14 with Stage III disease. When the patients were grouped according to the expression levels of each candidate gene, tumor stage was associated with ADAM15 expression (*p* = 0.0058), PPP1R14B expression (*p* = 0.0170), XPO5 expression (*p* = 0.0103), PPM1M expression (*p* = 0.0375), and FAM172A expression (*p* = 0.0006). FAM172A expression was also associated with ER status (*p* = 0.0363). No statistically significant association was detected between the expression of any candidate gene and age, PR status, or HER2 status in the available clinical data (Tables 1, 2, 3, 4, 5).

**TABLE 1:** Association between ADAM15 expression and clinicopathological characteristics in breast cancer patients (*n* = 56 cases).

Characteristics	Number	ADAM15 expression		*p*
		Low, no. cases	High, no. cases	
Age				
< 60	38	18	20	0.5671
≥ 60	18	10	8	
ER status				
Positive	46	21	25	0.1628
Negative	10	7	3	
PR status				
Positive	42	22	20	0.5371
Negative	14	6	8	
HER2 status				
Positive	17	11	6	0.1462
Negative	39	17	22	
Clinical stage				
I	23	17	6	0.0058
II	19	8	11	
III	14	3	11	

**TABLE 2:** Association between PPP1R14B expression and clinicopathological characteristics in breast cancer patients (*n* = 56 cases).

Characteristics	Number	PPP1R14B expression		*p*
		Low, no. cases	High, no. cases	
Age				
< 60	38	17	21	0.2524
≥ 60	18	11	7	
ER status				
Positive	46	22	24	0.4853
Negative	10	6	4	
PR status				
Positive	42	18	24	0.0641
Negative	14	10	4	
HER2 status				
Positive	17	11	6	0.1462
Negative	39	17	22	
Clinical stage				
I	23	16	7	0.0170
II	19	9	10	
III	14	3	11	

**TABLE 3:** Association between XPO5 expression and clinicopathological characteristics in breast cancer patients (*n* = 56 cases).

Characteristics	Number	XPO5 expression		*p*
		Low, no. cases	High, no. cases	
Age				
< 60	38	20	18	0.5671
≥ 60	18	8	10	
ER status				
Positive	46	24	22	0.4853
Negative	10	4	6	
PR status				
Positive	42	19	23	0.2170
Negative	14	9	5	
HER2 status				
Positive	17	8	9	0.7713
Negative	39	20	19	
Clinical stage				
I	23	17	6	0.0103
II	19	7	12	
III	14	4	10	

**TABLE 4:** Association between PPM1M expression and clinicopathological characteristics in breast cancer patients (*n* = 56 cases).

Characteristics	Number	PPM1M expression		*p*
		Low, no. cases	High, no. cases	
Age				
< 60	38	21	17	0.2524
≥ 60	18	7	11	
ER status				
Positive	46	21	25	0.1628
Negative	10	7	3	
PR status				
Positive	42	24	18	0.0641
Negative	14	4	10	
HER2 status				
Positive	17	9	8	0.7713
Negative	39	19	20	
Clinical stage				
I	23	7	16	0.0375
II	19	11	8	
III	14	10	4	

**TABLE 5:** Association between FAM172A expression and clinicopathological characteristics in breast cancer patients (*n* = 56 cases).

Characteristics	Number	FAM172A expression		*p*
		Low, no. cases	High, no. cases	
Age				
< 60	38	18	20	0.5671
≥ 60	18	10	8	
ER status				
Positive	46	20	26	0.0363
Negative	10	8	2	
PR status				
Positive	42	18	24	0.0641
Negative	14	10	4	
HER2 status				
Positive	17	11	6	0.1462
Negative	39	17	22	
Clinical stage				
I	23	5	18	0.0006
II	19	11	8	
III	14	12	2	

### 3.12 Cell‐Population‐Specific Expression of Candidate Genes

To examine the cellular distribution of the five candidate genes, their expression was measured in primary separated tumor cells, M1‐like TAMs, and M2‐like TAMs (Figure 12). ADAM15, PPP1R14B, and XPO5 had their highest expression in M1‐like TAMs. In contrast, FAM172A and PPM1M were expressed at lower levels in M1‐like TAMs and at higher levels in M2‐like TAMs.

**FIGURE 12:**
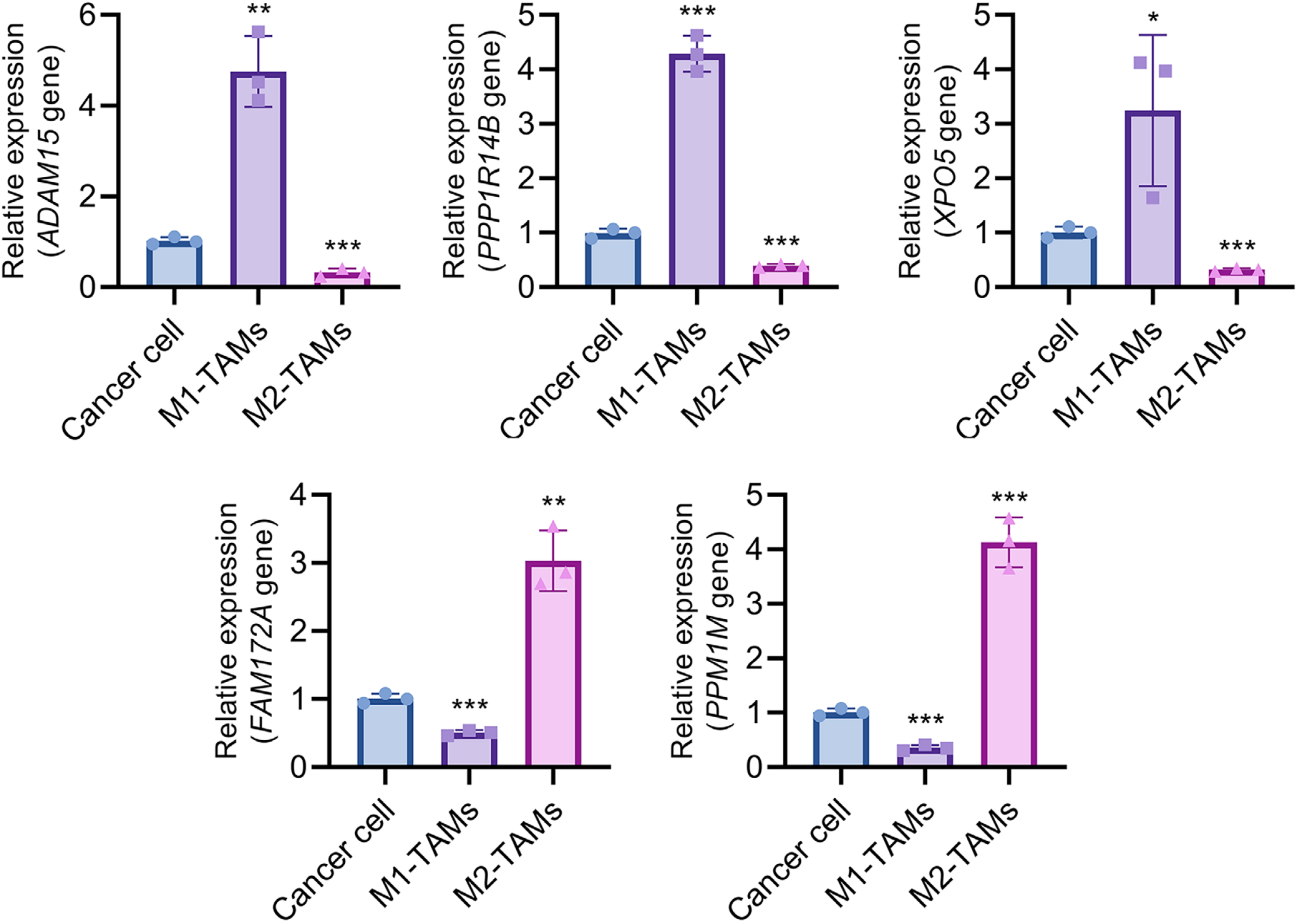
Expression of five candidate genes in separated tumor cells and tumor‐associated macrophage populations. The expression levels of ADAM15, PPP1R14B, XPO5, FAM172A, and PPM1M were analyzed by qRT‐PCR in primary tumor cells, M1‐like tumor‐associated macrophages (M1‐like TAMs), and M2‐like tumor‐associated macrophages (M2‐like TAMs).

### 3.13 Functional Effects of Candidate‐Gene Modulation in BMDMs

The functional relevance of the five candidate genes was further examined in BMDMs. ADAM15, XPO5, and PPP1R14B were overexpressed, whereas PPM1M and FAM172A were silenced; the corresponding OE‐CTRL and sh‐CTRL groups served as controls (Figure 13). Overexpression of ADAM15, XPO5, or PPP1R14B increased the proportion of CD80‐positive cells compared with OE‐CTRL. Similarly, knockdown of PPM1M or FAM172A increased the proportion of CD80‐positive cells relative to sh‐CTRL (Figure 13A,B). All five gene manipulations were also accompanied by higher relative ATP production, glucose uptake, and lactate production (Figure 13C–E).

**FIGURE 13:**
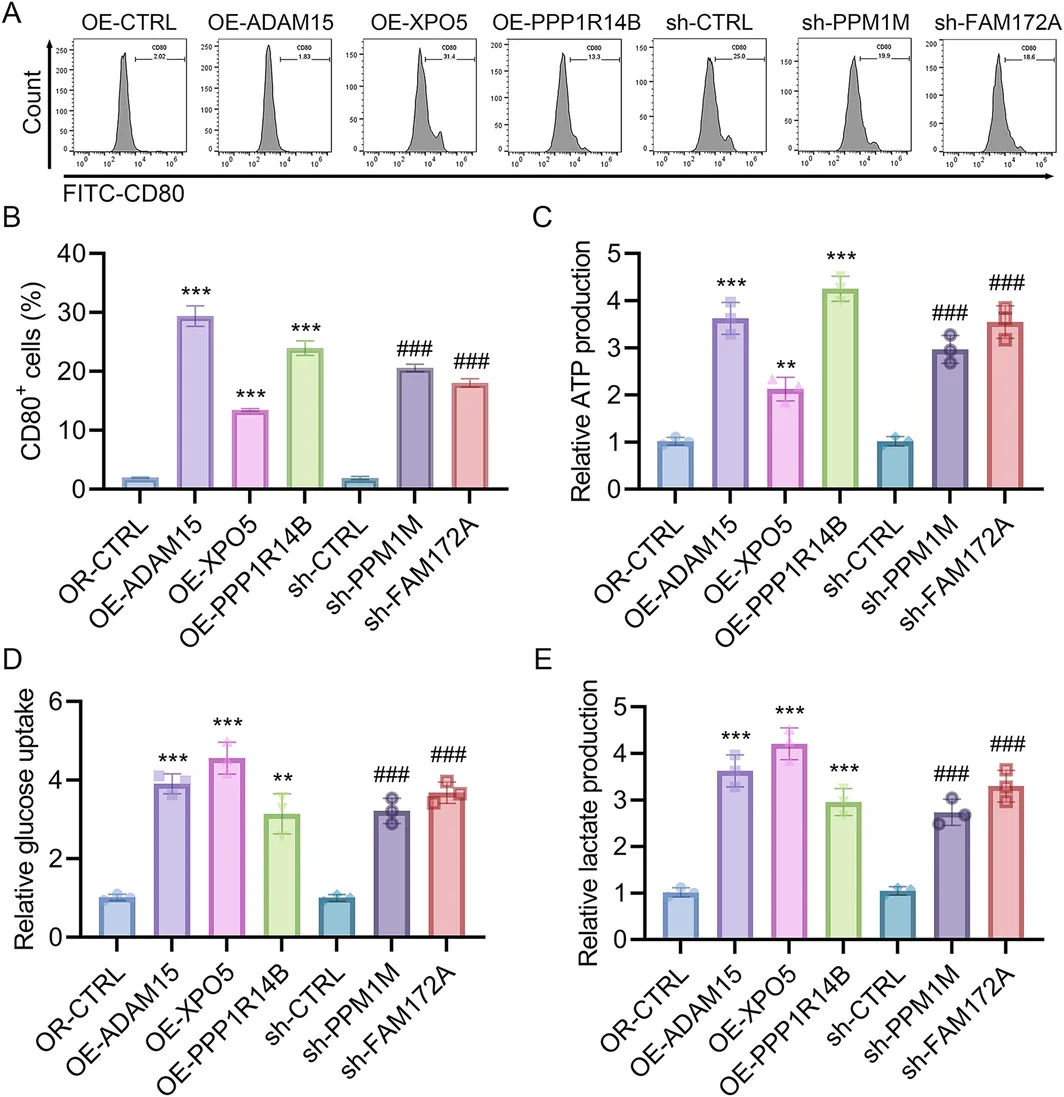
Effects of candidate‐gene modulation on macrophage polarization and glycolysis‐related metabolic parameters in BMDMs. The indicated genes were overexpressed (OE‐ADAM15, OE‐XPO5, and OE‐PPP1R14B) or silenced (sh‐PPM1M and sh‐FAM172A), with corresponding control groups. M1‐like polarization was assessed using CD80 expression and the proportion of CD80‐positive cells; ATP levels, glucose uptake, and lactate production were measured as glycolysis‐related metabolic readouts.

### 3.14 Cox Regression Analysis of Overall Survival

Univariate and multivariable Cox regression analyses were performed to further evaluate the prognostic associations of ADAM15, XPO5, PPP1R14B, PPM1M, and FAM172A (Figure 14). Gene‐specific hazard ratios, *p* values, and corresponding confidence intervals were obtained from both models and are presented in the forest plots.

**FIGURE 14:**
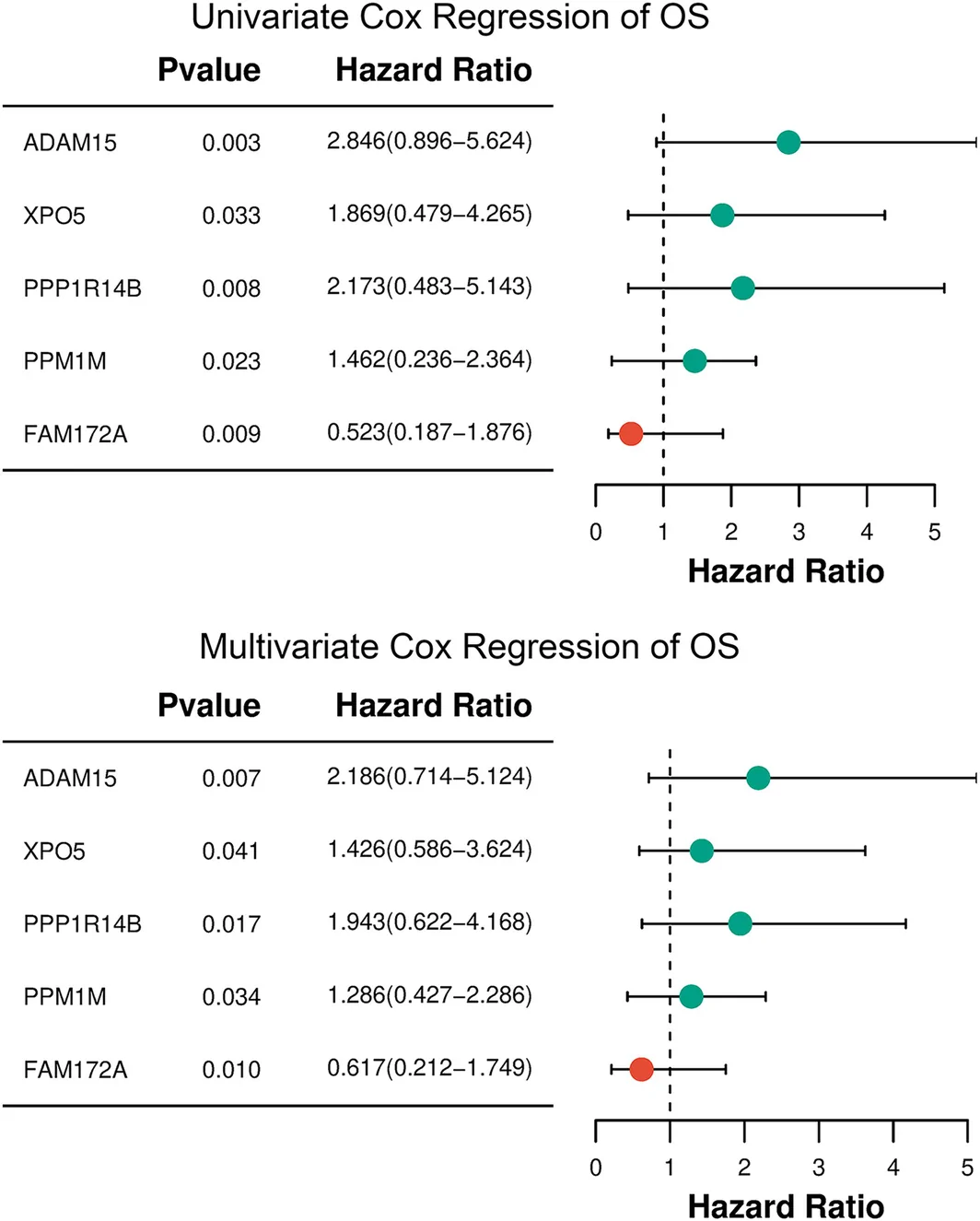
Univariate and multivariable Cox proportional hazards analyses of five candidate genes in BC. Forest plots present the hazard ratios (HRs) and corresponding confidence intervals for ADAM15, XPO5, PPP1R14B, PPM1M, and FAM172A in relation to overall survival.

## 4 Discussion

In this study, we examined glycolysis‐associated genes related to M1‐like macrophage features in breast cancer (BC) by integrating immune‐cell estimation, transcriptomic analysis, weighted gene co‐expression network analysis (WGCNA), Mendelian randomization (MR), and machine‐learning‐based feature selection. We identified 101 genes that overlapped among the M1 macrophage‐associated, glycolysis‐related, and differentially expressed gene sets. Eight genes showed suggestive associations with BC risk in MR analysis, seven genes were retained as diagnostic candidates, and five genes were further examined in clinical tissues, separated cell populations, and bone marrow‐derived macrophages (BMDMs). This analysis led us to examine whether the selected genes retained diagnostic and survival‐related signals in additional datasets and experimental models. The tissue, cell‐sorting, and BMDM experiments addressed the cellular source and functional association of these candidates, but did not show that they mediate tumor–macrophage metabolic crosstalk.

A major finding was the higher M1‐like macrophage signal observed in BC samples. This pattern was detected as increased CIBERSORT‐derived M1‐like macrophage proportions and xCell‐derived M1‐like enrichment scores. TAMs in BC have often been described as predominantly M2‐like and associated with tumor progression; however, current evidence indicates that TAMs do not form two fixed and mutually exclusive populations. Instead, they occupy a continuum of activation states shaped by local cytokines, metabolites, spatial location, and interactions with tumor and stromal cells (Choi et al. 2018; Mehta et al. 2021). Therefore, the present results should be interpreted as an increase in an M1‐like transcriptional signal rather than direct evidence of a uniform M1 macrophage population. Using the xCell‐derived M1‐like score as a trait, WGCNA identified 2374 genes associated with this bulk‐tissue expression pattern. These genes provided a basis for examining the relationship between macrophage‐associated signals and tumor metabolism, but they cannot by themselves define a macrophage‐specific transcriptional program.

The overlap between M1‐like macrophage‐associated genes and glycolysis‐related genes is consistent with the metabolic changes that commonly accompany inflammatory macrophage activation. M1‐like macrophages commonly favor aerobic glycolysis during inflammatory responses, although glycolytic and oxidative pathways are not restricted to a single macrophage state (Dussold et al. 2024). In BC, tumor‐derived lactate has been shown to alter inflammatory macrophage polarization and reduce M1‐associated features (Wang et al. 2023). These observations support the biological plausibility of a relationship between tumor metabolism and macrophage state. In our study, glycolysis scores were higher in BC tissues, and 101 genes were identified at the intersection of glycolysis‐related, M1‐like macrophage‐associated, and differentially expressed genes. This gene set should be regarded as a candidate pool for studying immune–metabolic relationships rather than as a confirmed molecular signature or a defined mechanistic pathway.

To prioritize candidates using genetic evidence, we performed two‐sample MR analysis and identified eight genes with suggestive associations between genetically predicted expression and BC risk, including ADAM15, ALAS1, FAM172A, PPM1M, PPP1R14B, TP53BP2, XPO5, and ZNF318. ADAM15 has been associated with aggressive breast cancer and metastatic progression (Kuefer et al. 2006). XPO5 participates in the nuclear export of precursor miRNAs and has been linked to breast tumorigenesis (Leaderer et al. 2011), although its relationship with macrophage‐associated immune phenotypes remains unclear. PPM1M has mainly been investigated in pan‐cancer analyses, where its expression has been related to immune features and prognosis, but its specific role in BC has not been established (Zeng et al. 2023). FAM172A has been recognized as a tumor suppressor in colorectal cancer (Cui et al. 2016), but limited evidence supports its function in the context of BC. PPP1R14B also requires careful interpretation because a previous study in triple‐negative breast cancer (TNBC) linked its upregulation to STMN1 stabilization, tumor progression, and paclitaxel resistance (Liao et al. 2023). This observation differs from the favorable survival association observed for PPP1R14B in our analysis and suggests that its effects may depend on molecular subtype, treatment context, or cellular compartment. The combination of differential expression, immune associations, pathway enrichment, and MR‐based genetic evidence was useful for candidate prioritization and hypothesis generation, but does not establish a biological mechanism. MR estimates reflect lifelong genetically proxied differences in gene expression at the population level and depend on the validity of the instrumental‐variable assumptions. They may not capture acute, cell‐type‐specific, or context‐dependent changes within the BC tumor microenvironment (Lu et al. 2024; Nguyen and Mitchell 2024).

Building on these findings, we used LASSO and SVM‐RFE to derive a seven‐gene diagnostic panel. In the GSE65194 discovery cohort, each of the seven genes showed an AUC > 0.7 for distinguishing BC from non‐tumor tissues. These gene‐wise AUCs indicate discriminatory performance in the discovery dataset, but they do not constitute prospective clinical validation of a combined seven‐gene model. Five genes showed concordant tumor–normal expression changes in TCGA‐BRCA. ALAS1 changed in the opposite direction, and ZNF318 was not significantly different. In the 56 paired clinical tissues, ADAM15, PPP1R14B, and XPO5 were increased in tumors, whereas FAM172A and PPM1M were decreased. The single‐gene AUCs in this cohort ranged from 0.6014 for XPO5 to 0.8559 for ADAM15, indicating that the diagnostic contribution of individual genes was not uniform. Only five of the seven diagnostic genes were evaluated in the 56‐patient tissue cohort, and the combined seven‐gene model was not independently tested in this cohort. FAM172A and PPP1R14B may be less well characterized in the specific context of glycolysis‐associated M1‐like macrophage biology in BC, but their inclusion should not be interpreted as evidence that they are completely novel BC genes. Overall, the seven‐gene model should be considered a discovery‐stage classifier that requires testing in independent, prospective, and preferably multicenter cohorts (Efthimiou et al. 2024).

The survival analysis showed associations for all five genes examined in the prognostic analysis. Higher ADAM15, FAM172A, and XPO5 expression was associated with shorter overall survival, whereas higher PPM1M and PPP1R14B expression was associated with longer overall survival. In the current analysis, the multivariable Cox model included the five candidate genes but did not fully adjust for major clinicopathological variables such as tumor stage, grade, receptor status, age, and treatment history. These results should therefore be interpreted as associations estimated from a model containing the five genes, rather than evidence that the genes are independent prognostic factors. The clinical tissue cohort also showed stage‐related differences in the expression of all five genes, and FAM172A expression was associated with ER status, supporting the possibility that clinical and molecular characteristics contributed to the observed survival patterns. The opposite directions of association may reflect differences in tumor subtype, treatment exposure, cellular source, or the relationship between bulk‐tissue expression and macrophage‐specific expression. In particular, ADAM15, PPP1R14B, and XPO5 were most highly expressed in M1‐like TAMs, whereas FAM172A and PPM1M were more highly expressed in M2‐like TAMs; bulk‐tissue survival signals therefore integrate multiple cellular compartments and should not be equated with macrophage‐specific effects. The favorable association of PPP1R14B with survival in our cohort also contrasts with its reported role in TNBC progression and paclitaxel resistance (Liao et al. 2023). This discrepancy cannot be resolved by the current data and should be examined in subtype‐specific cohorts with detailed treatment information.

The additional experiments addressed the cellular source and functional context of the transcriptomic findings. In paired tissues, the tumor‐associated expression differences of the five candidates were reproduced. In separated primary cell populations, ADAM15, PPP1R14B, and XPO5 showed the highest expression in M1‐like TAMs, whereas FAM172A and PPM1M were more highly expressed in M2‐like TAMs. This result supports cell‐population‐specific expression patterns and also illustrates why associations derived from bulk tissue should not be interpreted as uniformly M1‐specific. In BMDMs, overexpression of ADAM15, XPO5, or PPP1R14B, as well as knockdown of PPM1M or FAM172A, was accompanied by increased CD80 positivity, ATP levels, glucose uptake, and lactate production. These findings are compatible with a relationship between the candidate genes and a CD80‐positive, glycolysis‐related macrophage phenotype. However, CD80 represents only one macrophage activation marker, and ATP, 2‐NBDG uptake, and lactate production are indirect metabolic readouts rather than direct measurements of glycolytic flux. The experiments also did not include tumor‐cell co‐culture, rescue experiments, or in vivo validation. Thus, they support functional relevance in macrophages but do not establish a complete tumor–macrophage mechanism in BC.

The TF–miRNA–mRNA analysis generated predicted regulatory relationships involving the five candidate genes. These interactions may help identify regulatory hypotheses for future experiments, but their activity and relevance to M1‐like macrophage biology were not directly tested. The five genes do not currently define a single demonstrated linear pathway. A working model is that they capture complementary components of an immune–metabolic state, including extracellular‐matrix and adhesion‐related pathways, metabolic processes, and putative miRNA‐related regulation. This model is hypothesis‐generating because the current analyses were performed largely in bulk tissue and did not test the connectivity among these modules. The observed functional diversity may explain the different expression and survival patterns across the genes and suggests that the panel may represent a composite set of biomarkers rather than a single mechanistically unified axis.

Single‐gene GSEA further supported this functional heterogeneity. ADAM15 was associated with ECM–receptor interaction and focal adhesion, FAM172A with oxidative phosphorylation, PPM1M with ECM–receptor interaction, PPP1R14B with focal adhesion, and XPO5 with TCA‐cycle and proteasome‐related pathways. These findings suggest that the candidate genes may capture several tumor‐associated and immune‐associated transcriptional programs. However, expression‐based GSEA indicates differential pathway enrichment and does not demonstrate pathway activation, identify the responsible cell type, or establish a causal relationship. The enriched pathways should therefore be considered hypotheses for subsequent cell‐type‐specific and mechanistic studies.

This study has several limitations. First, the public cohorts were retrospective and generated using different transcriptomic platforms. GSE65194 and TCGA‐BRCA were normalized separately and were not combined into a single expression matrix, which avoids direct mixing of microarray and RNA‐sequencing values but does not eliminate platform‐specific or cohort‐specific bias. TCGA‐BRCA was used as an external expression reference and for exploratory survival analysis, but it was not a prospective or multicenter validation cohort and should not be regarded as an independent prospective validation of the complete classifier. The seven‐gene model was not tested prospectively in an independent clinical population, and the diagnostic performance of individual genes in the 56‐patient cohort was variable. In addition, subtype‐stratified survival analysis was not performed for the prognostic genes or the diagnostic model. Given the biological heterogeneity of luminal, HER2‐positive, and triple‐negative BC, the observed associations may not apply equally across molecular subtypes (Xiong et al. 2025). The survival model used median expression cutoffs, and alternative cutoffs or time‐dependent performance were not evaluated. Calibration and decision‐curve estimates derived from the discovery data may also be optimistic despite bootstrap bias correction.

Furthermore, immune‐cell composition was estimated from bulk transcriptomic data using CIBERSORT and xCell. Such methods provide relative proportions or enrichment scores rather than direct cell counts (Newman et al. 2015; Aran et al. 2017), and their performance can be affected by tumor purity, related cell populations, and sample heterogeneity (Tran et al. 2023). The separation of primary tumor cells and macrophage populations reduced, but did not eliminate, the concern that all qRT‐PCR signals originated from total tissue RNA. The marker‐based definition of M1‐like and M2‐like TAMs cannot fully represent the continuous and mixed phenotypes of macrophages. The BMDM experiments used a murine macrophage model, tested one perturbation direction for each gene, and did not include a time‐course analysis of cytokine‐induced macrophage activation. Because the expression analyses were cross‐sectional and the BMDM assays were endpoint experiments, the temporal changes of these genes during macrophage activation or BC progression cannot be determined. Direct glycolytic flux measurements, tumor–macrophage co‐culture, rescue experiments, human macrophage models, and in vivo studies will be needed to determine whether these genes participate in a causal metabolic interaction within BC. Finally, the MR findings depend on the validity of the instrumental‐variable assumptions and may not reflect the short‐term, tissue‐specific biology of the BC tumor microenvironment (Nguyen and Mitchell 2024). The regulatory network and GSEA results are also predictive and require experimental confirmation.

## 5 Conclusions

This study integrated transcriptomic, genetic, and experimental analyses to identify glycolysis‐associated genes related to M1‐like macrophage characteristics in BC. All seven selected genes distinguished tumor from normal tissue in GSE65194; five showed concordant expression changes in TCGA‐BRCA. Five candidates were further examined in paired clinical tissues, separated cell populations, and BMDMs. The tissue and cellular findings, together with changes in CD80 and glycolysis‐related metabolic readouts after gene manipulation, provide preliminary support for an association between these genes and macrophage immune–metabolic phenotypes. However, the current data do not establish tissue‐specific causality, mediation of tumor–macrophage crosstalk, or independent prognostic value. Subtype‐stratified prospective validation and mechanistic studies involving tumor–macrophage co‐culture, direct metabolic‐flux measurements, rescue experiments, and in vivo models are required before clinical translation.

## Author Contributions

**Liang Zhang:** investigation, data curation, writing – original draft, methodology, conceptualization. **Lingyuan Wu:** data curation, formal analysis. **Can Xu:** data curation, validation. **Chao Fang:** investigation, methodology. **Xingsong Tian:** data curation, visualization. **Kunbing Zhu:** resources, funding acquisition, writing – review and editing.

## Funding

This research was funded by the Shandong Province Medical and Health Science and Technology Development Project (Nos 20250411226, 2019WS424) and the Special Fund Project of Breast Disease Research of Shandong Medical Association (No. YXH2021ZX052) and the Shandong University Industry‐sponsored Research Project (No. 12721742) and the Shandong Provincial Maternal Child Health Youth Science and Technology Innovation Special Project (No. SFYZXJJ‐2024036).

## Ethics Statement

All procedures performed in this study were approved by the Research Ethics Committee of Shandong Province Maternal and Child Health Care Hospital (No. 2025–031) and with the Declaration of Helsinki (as revised in 2013) and relevant national regulations. Written informed consents were obtained from patients.

## Consent

The authors have nothing to report.

## Conflicts of Interest

The authors declare no conflicts of interest.

## Supporting information


**Table S1:** Baseline clinicopathological characteristics of three study cohorts.

## Data Availability

The datasets used and/or analyzed during the current study are available from the corresponding author upon reasonable request.
