# Supplementary material for: Analysis and Identification of Candidate Genes Associated with M1 Macrophage Infiltration and Glycolysis in Breast Cancer
Source: Chem Biol Drug Des. 2026 Nov 5;108(5):e70437. doi: 10.1111/cbdd.70437 (PMC13740340; doi:10.1111/cbdd.70437)
Supplement: Supplementary file 1 [file CBDD-108-e70437-s001.docx]

Supplementary Table S1 Baseline clinicopathological characteristics of three study cohorts

| **Characteristic** | **GSE65194** | | **TCGA-BRCA** | | **Hospital cohort Patients (n = 56)** |
| --- | --- | --- | --- | --- | --- |
|  | **Normal tissue (n = 11)** | **Breast cancer (n = 130)** | **Normal tissue (n = 113)** | **Breast cancer (n = 1,095)** |  |
| Age, years | — | 54.15 ± 12.49 | 57.93 ± 14.51 | 58.45 ± 13.22 | — |
| Age <60 years, n (%) | — | — | — | — | 38 (67.9) |
| Age ≥60 years, n (%) | — | — | — | — | 18 (32.1) |
| Female, n (%) | — | — | 112 (99.1) | 1,083 (98.9) | — |
| Tumor size, mm | — | 20.0 (15.0–30.0) | — | — | — |
| Stage I, n (%) | — | — | — | 183 (16.7) | 23 (41.1) |
| Stage II, n (%) | — | — | — | 622 (56.8) | 19 (33.9) |
| Stage III, n (%) | — | — | — | 249 (22.7) | 14 (25.0) |
| Stage IV, n (%) | — | — | — | 20 (1.8) | — |
| Stage unknown, n (%) | — | — | — | 21 (1.9) | — |
| Subtype: Luminal A, n (%) | — | 29 (22.3) | — | — | — |
| Subtype: Luminal B, n (%) | — | 30 (23.1) | — | — | — |
| Subtype: HER2, n (%) | — | 30 (23.1) | — | — | — |
| Subtype: Basal-like, n (%) | — | 41 (31.5) | — | — | — |
| ER-positive, n (%) | — | 59 (45.4) | — | — | 46 (82.1) |
| ER-negative, n (%) | — | — | — | — | 10 (17.9) |
| PR-positive, n (%) | — | 44 (33.8) | — | — | 42 (75.0) |
| PR-negative, n (%) | — | — | — | — | 14 (25.0) |
| HER2-positive, n (%) | — | 54 (41.5) | — | — | 17 (30.4) |
| HER2-negative, n (%) | — | — | — | — | 39 (69.6) |
| Survival observation time, months | — | 49.1 (31.9–70.0) | — | — | — |
| Deaths, n (%) | — | 18 (13.8) | — | — | — |

**Notes.** Values are mean ± standard deviation, median (interquartile range), or n (%), as applicable. Stage denotes pathological stage in TCGA-BRCA and clinical stage in the hospital cohort. Hospital-cohort percentages use 56 patients as the denominator. In the normal-tissue columns, — indicates not applicable for tumor-specific characteristics; elsewhere, — indicates information not reported in the cohort descriptions used for this table. ER, estrogen receptor; PR, progesterone receptor; HER2, human epidermal growth factor receptor 2.
